# REM sleep behavior in Parkinson disease: Frequent, particularly with higher age

**DOI:** 10.1371/journal.pone.0243454

**Published:** 2020-12-07

**Authors:** Heide Baumann-Vogel, Hyun Hor, Rositsa Poryazova, Philipp Valko, Esther Werth, Christian R. Baumann

**Affiliations:** Department of Neurology, University Hospital and University of Zurich, Zurich, Switzerland; Universitat Ulm, GERMANY

## Abstract

This retrospective single-center polysomnography-based study was designed to assess the frequency of REM sleep behavior disorder (RBD) in consecutive patients with Parkinsonism, including Parkinson disease, dementia with Lewy bodies, multiple system atrophy, progressive supranuclear palsy, and corticobasal degeneration. We observed RBD in 77% of 540 Parkinson patients, with rising frequency at higher age and regardless of sex, in >89% of 89 patients with dementia with Lewy bodies or multiple system atrophy, and in <15% of 42 patients with progressive supranuclear palsy or corticobasal degeneration. Thus, the prevalence of RBD in sporadic Parkinson disease might be higher than previously assumed, particularly in elderly patients.

## Introduction

Rapid eye movement (REM) sleep behavior disorder (RBD) is characterized by REM sleep without atonia in conjunction with recurrent nocturnal dream enactment [1]. It constitutes a prodromal biomarker, as the presence of isolated RBD is a herald of imminent neurodegenerative disease, particularly α-synuclein-related Parkinsonism which includes PD, multiple system atrophy (MSA), and dementia with Lewy bodies (DLB) [2–4]. In addition, RBD in PD patients is associated with faster progression of both motor and non-motor symptoms [5–7]. The clinical significance of RBD is reflected by the fact that it became a core clinical feature of DLB [8]. In contrast, the current clinical diagnostic criteria for PD remain centered on a defined motor syndrome, yet the absence of RBD and other common non-motor symptoms despite 5 years disease duration is considered a red flag [9].

The studies on RBD prevalence in PD, however, are inconclusive as they report a wide range of frequencies, which is most probably owed to different methods for RBD screening, and to the inclusion of variable populations. A recent review summarized substantial literature which states that roughly half of PD patients have REM sleep without atonia or RBD [10]. Another review reported that the prevalence of RBD in PD patients followed by tertiary centers and based on polysomnography recordings is around 39–46% [11]. A meta-analysis in 2462 PD patients assessed RBD by means of either polysomnography, structured questionnaire, codes in patient medical records, or medication used to treat RBD symptoms, and found an overall prevalence of 23.6% [12]. On the other hand, a recent cross-sectional study examined polysomnography findings of 88 Parkinson patients and found an RBD prevalence of 62.5% [13].

We conducted a retrospective study of our database of sleep recordings in patients with movement disorders, to assess the prevalence of RBD in PD and related disorders (DLB, MSA, progressive supranuclear palsy, PSP, or corticobasal degeneration, CBD).

## Methods

This is a retrospective study including all patients with PD, DLB, MSA, progressive supranuclear palsy (PSP), and corticobasal degeneration (CBD), who underwent at least one polysomnography recording in the sleep laboratory of the Department of Neurology, University Hospital Zurich, between October 2002 and July 2019. The data were analyzed anonymously, and the study was approved by the local ethics committee ("Kantonale Ethikkommission Zürich", 2014–0127). Patients have provided written informed consent beforehand to allow data from their medical records to be used in our research.

Due to the close clinical and scientific interaction of the sleep and movement disorders units within the department, more than 90% of patients with neurodegenerative disease underwent polysomnography, irrespective of the suspected presence of RBD or other sleep-wake disturbances. Diagnoses in all patients were re-checked against current diagnostic criteria [8, 9, 14–16], and those with Parkinsonism but without an established diagnosis were not included in this analysis (n = 29). In addition, we classified PD patients into three subgroups: PD patients with young onset (first motor symptoms before the age of 40 years), and patients with either akinetic-rigid or tremor-dominant PD, as introduced before [17]. We recorded sex, age, and disease duration at the time of polysomnography in all patients. Patients with depression or insomnia as a comorbidity were asked to pause antidepressant and hypnotics treatment at least 2 days before polysomnography.

Single-night video-polysomnography recordings were performed as described previously [18]: Sleep was scored along Rechtschaffen and Kales (until 2007) and along AASM guidelines (from 2007 onwards) [19, 20]. Features of RBD were explored based on EMG recordings on chin, anterior tibialis and upper extremities (from 2010 onwards) and time-synchronized, audio-equipped video. We diagnosed RBD if one of the two following situations was fulfilled:

- Polysomnography showed unequivocal acting out of dreams (motor/emotional behavior, vocalization) during REM sleep + REM sleep with impaired atonia.
- Consistent report of a bedpartner of dream enactment in the second half of the night + REM sleep with impaired atonia during polysomnography.

REM sleep with impaired atonia was diagnosed based on experts’ opinion by either or both of the following features: sustained muscle activity in REM sleep in the chin EMG and/or excessive transient muscle activity during REM sleep in the chin or limb EMG. All polysomnography scorings were supervised by one of the six authors. A quantification cut-off, as introduced by other groups for defining RBD in polysomnography, had not been used in most recordings in our sleep laboratory ever since 2002, hence this data was not available for this retrospective study [21, 22].

## Results

We included 671 patients, whereof 540 patients with PD (mean age: 71.6 years; 326 with akinetic-rigid PD, 151 with tremor-dominant PD, and 63 with young-onset PD), 28 patients with DLB, 61 patients with MSA, 35 patients with PSP, and 7 patients with CBD (Table 1). Detailed results are presented in Table 1. RBD prevalence in the entire PD cohort was 76.9%, with much lower frequencies in young-onset patients (46%). Prevalence was higher in MSA (91.8%) and DLB patients (89.3%), but much lower in PSP (11.4%) and CBD patients (14.3%). In PD patients, the two genders were equally affected. Bed partners reported acting out dreams in 198 PD patients, while PSG confirmed RBD in 167 of these patients. Among the 125 PD patients without RBD (23.1%) in our sample, we counted 91 patients with non-young-onset Parkinson disease without RBD. Twelve patients of this group (13%) showed absent or very short (<15 minutes) REM sleep. Among the remaining 34 young-onset PD patients without RBD, we found only one patient (3%) with very short (9 minutes) REM sleep.

**Table 1:** Prevalence of RBD in neurodegenerative Parkinsonism.

	All	Men	Women
**Parkinson disease**	415/540 (**76.9%**)	259/337 (**76.9%**)	156/203 (**76.8%**)
***akinetic-rigid***	275/326 (**84.4%**)	161/187 (**86.1%**)	114/139 (**82.0%**)
***tremor-dominant***	111/151 (**73.5%**)	75/101 (**74.3%**)	36/50 (**72.0%**)
***young-onset***	29/63 (**46.0%**)	23/49 (**46.9%**)	6/14 (**42.9%**)
**Dementia with Lewy bodies**	25/28 (**89.3%**)	20/23 (**87.0%**)	5/5 (**100.0%**)
**Multiple system atrophy**	56/61 (**91.8%**)	30/33 (**90.9%**)	26/28 (**92.9%**)
**Progr. supranuclear palsy**	4/35 (**11.4%**)	3/17 (**17.6%**)	1/18 (**5.6%**)
**Corticobasal degeneration**	1/7 (**14.3%**)	0/3 (**0%**)	1/4 (**25.0%**)

**Fig 1:**
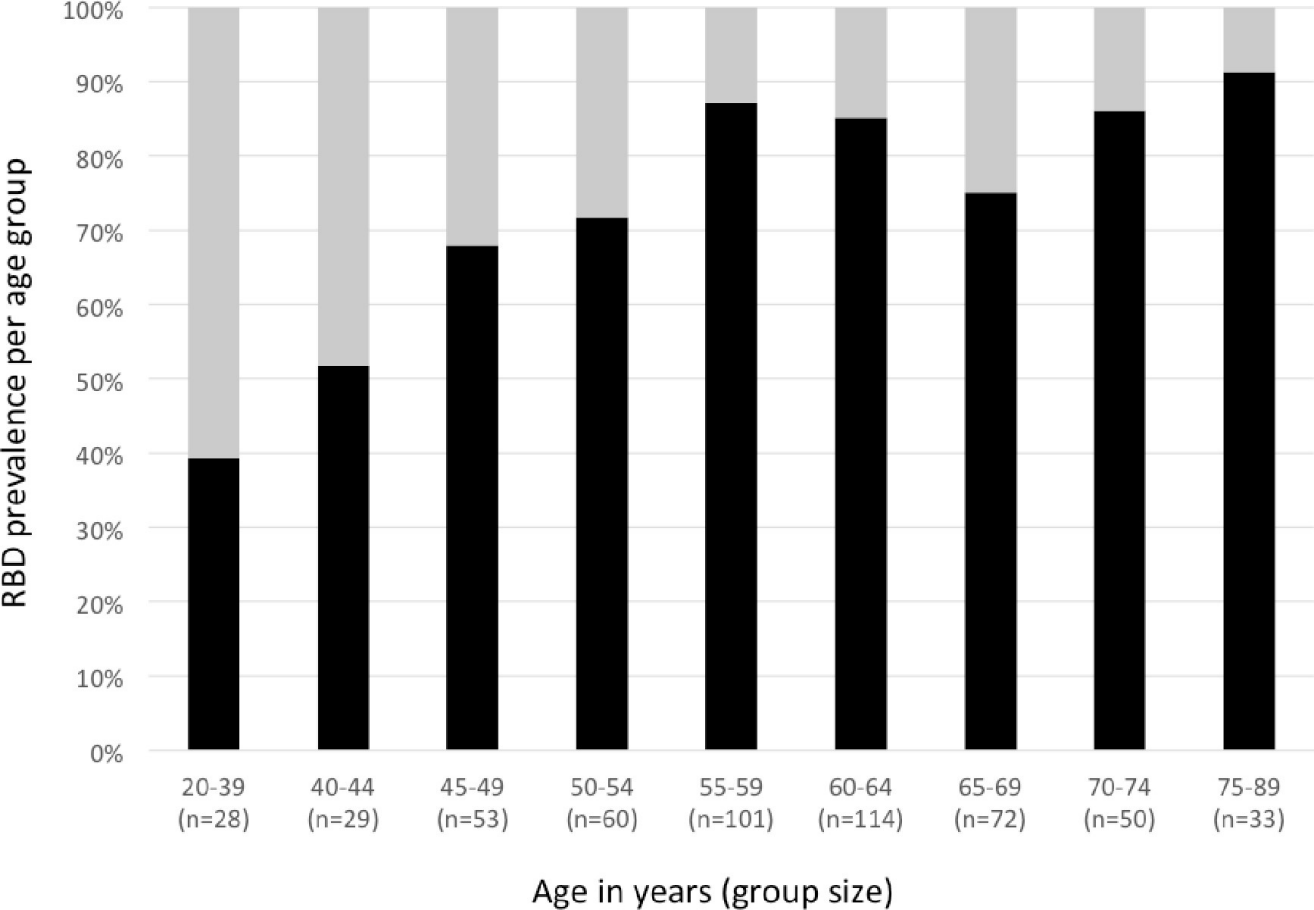
Prevalence of RBD (black segments) in the entire Parkinson patient sample (n = 540) across age groups (in years).

In PD patients, disease duration at time of polysomnography (8.4±4.9 years, mean±standard deviation) was not different between patients with and without RBD, but pooled akinetic-rigid and tremor-dominant PD patients with RBD were significantly older than those without RBD (65.8±8.4 vs. 60.7±10.7 years, p = 0.01). Also, prevalence of RBD in the entire PD population rose with age (Fig 1). In the 236 PD patients with repeated polysomnography, the prevalence of RBD was 82.6% (195/236 patients). In these patients, the mean interval between polysomnographies was 15.7 months (range 6–62 months). We observed RBD in both polysomnographies in 146 patients, only in the first recording in 14 patients, and only in the second recording in 35 patients. Thus, in 49 of 195 (25%) PD patients with RBD and repeated polysomnography, RBD was diagnosed in only one of two recordings. In all 14 patients who presented RBD only in the first recording, we noted absent or very short REM sleep in the second, RBD-negative night. A closer look at the 35 patients in whom we observed RBD exclusively in the second recording, revealed five young onset PD patients in whom long episodes of REM sleep were observed already in the first recording.

## Discussion

The goal of this retrospective and descriptive study was to assess the prevalence of RBD in a large set of patients with neurodegenerative Parkinsonism examined by video-polysomnography. We found a much higher than previously reported prevalence of RBD in PD patients (77% of 540 patients), particularly in non-tremor-dominant PD patients whose disease onset was beyond the age of 40 years (84%), increasing with age. The frequency of RBD in other neurodegenerative disorders confirmed the clear association with α-synuclein (DLB: 89%, and MSA: 92%, versus PSP: 11%, and CBD: 14%) [2–4].

The reason for this higher than previously reported prevalence might be explained by several factors: first of all, we took advantage of our long-term experience and expertise in diagnosing both PD and RBD. Second, the use of the REM sleep without atonia quantification cut-off as introduced by other groups for defining RBD in polysomnography might deliver lower frequencies [21, 22]. Indeed, in a previous analysis in a smaller cohort of PD patients before and after deep brain stimulation, we found a positive REM sleep without atonia index above the SINBAR cutoff in only 65% of the patients [18, 22]. Third, we included a large portion of patients with repeated polysomnography recordings, which additionally increased prevalence. Fourth, the mean age of our cohort (71.6 years) was higher than in previous studies (i.e. 67.4 or 60.8) [13, 23], again corroborating our additional finding of increased prevalence with rising age, though sample sizes were much lower in previous studies. A factor that might have erroneously increased prevalence was the inclusion of patients on antidepressant treatment. Although this medication had been discontinued at least 2 days before PSG, we should keep in mind that long-term effects cannot be excluded with an ongoing impact on REM sleep atonia. However, there are previous studies suggesting that antidepressants may “unmask” RBD rather than cause it [24]. We therefore decided to avoid exclusion of PD patients on antidepressant treatment to ensure the use of an unbiased cohort.

As reported before, the prevalence of RBD was higher in akinetic-rigid PD patients compared to those with tremor-dominant PD [25]. Findings on the association of RBD with gender have been inconsistent so far, with some authors finding RBD predominantly in men [26–28]. In our sample, we found no differences of RBD frequency between women and men. Also, RBD prevalence was independent of disease duration, in contrast with earlier observations, though among non-young onset PD patients, those with RBD were older than those without RBD, but disease duration was still not different [28]. This suggests—in accordance with earlier studies—that the prevalence of RBD rises with age [29, 30]. This also reflects the high likelihood of developing RBD when getting older, i.e. the lifetime prevalence of RBD in PD patients. In young-onset PD patients, RBD was much less frequent, as discussed already before [31].

The retrospective setting of this study has the advantage that the patients were unselected, yet we identify two limitations: First, the recommended diagnosis of RBD and related polysomnography procedures were adapted during this time [21, 22]. We therefore chose a conservative approach to diagnose RBD as introduced above, by requiring reliable evidence of dream enactment. Second, an inclusion bias cannot be entirely excluded. However, due to our long-term interest in the association of sleep with neurodegenerative disease with recording sleep in a vast majority of neurodegeneration patients—reliable figures are available from 2008 to 2019, where 92% of patients with neurodegenerative Parkinsonism from the movement disorders unit were recorded in the sleep lab—we are confident that such an inclusion bias should be minimal.

Together with the positive response to levodopa, and along with neuroimaging such as positive DaT-SPECT imaging or typical substantia nigra hyperechogenicity in transcranial sonography [32, 33], polysomnography could be a useful tool in the differential diagnosis of PD. If present, RBD is a strong but not 100% specific indicator of a synuclein-related disorder. However, negative findings cannot exclude PD, particularly in younger Parkinsonism patients. In addition, the current study revealed that a single polysomnography could miss RBD in a significant portion of PD patients (here: in 25%). This might reflect insufficient sleep or missing REM sleep during polysomnography, but might also partially be explained by the fact that we did not aim to identify patients with isolated REM without atonia, but rather patients with RBD. More importantly, this finding could indicate RBD development between two recordings, i.e. with older age, and further strengthen recent hypotheses on the spatial progression of PD pathology [34]. Hence, repeated polysomnography measurements might help to understand potential subtypes and pathological mechanisms in PD.

Altogether, RBD is a highly prevalent sleep disorder not only in MSA and DLB, but also in sporadic PD, particularly in older patients, underlining its tight association with all kinds of synuclein disorders, and its diagnostic and prognostic role both in prodromal and manifest disease.
